# Supplementary material for: Solar photovoltaic interventions have reduced rural poverty in China
Source: Nat Commun. 2020 Apr 23;11:1969. doi: 10.1038/s41467-020-15826-4 (PMC7181783; doi:10.1038/s41467-020-15826-4)
Supplement: Supplementary file 1 — Supplementary Information [file 41467_2020_15826_MOESM1_ESM.pdf]

Supplementary Information

Solar Photovoltaic Interventions Have Reduced Rural Poverty in China

Zhang et al.

Contents

Supplementary Figure 1. Parallel trend after matching
Supplementary Figure 2. Kernel density of treatment and control group
Supplementary Figure 3. Number of counties by type in our sample
Supplementary Table 1. Partial list of factors affecting solar deployment or solar poverty alleviation
Supplementary Table 2. Summary statistics
Supplementary Table 3. Correlation matrix
Supplementary Table 4. Variance inflation factor test
Supplementary Table 5. Alternative definitions and regression specifications
Supplementary Table 6. Alternative definitions and regression specifications (excluding national poor counties)
Supplementary Table 7. The effect of PV policy on rural per capita disposable income (parallel trend test)
Supplementary Table 8. The effect of PV policy on rural per capita disposable income (sub-samples)
Supplementary Table 9. The effect of PV policy on substitution dependent poverty variables
Supplementary Table 10. The effect of PV policy on raw rural per capita disposable income
Supplementary Table 11. Endogenous treatment effect
Supplementary Note 1. Robustness check- Subsample analysis
Supplementary Note 2. Robustness check- Measures of dependent variables
Supplementary Note 3. Robustness check- Endogenous treatment effect

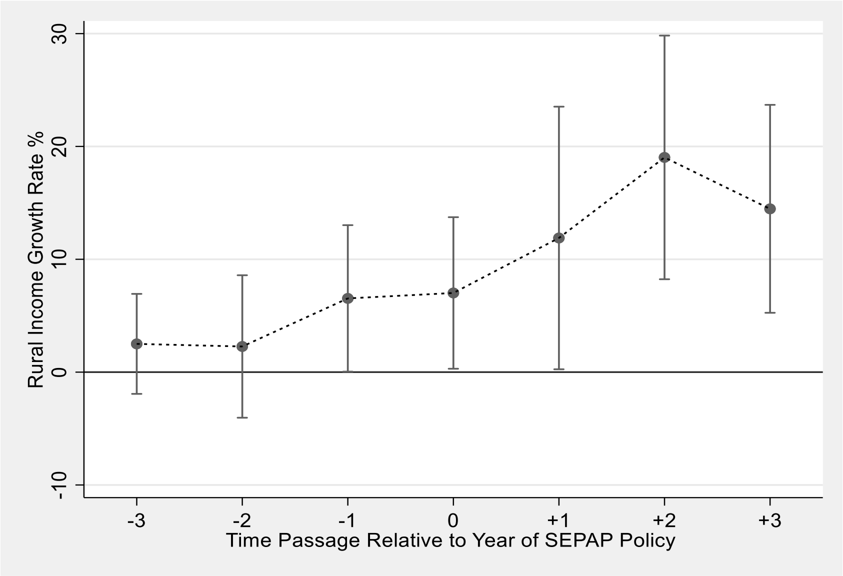

Supplementary Figure 1. Parallel trend after matching: a. Illustration of annual difference in the growth rate of rural income per capita before and after treatment (PV poverty alleviation policy) to demonstrate pre-treatment parallel trend assumption and post-treatment measured effect. b. Source data are provided in the Source Data file.

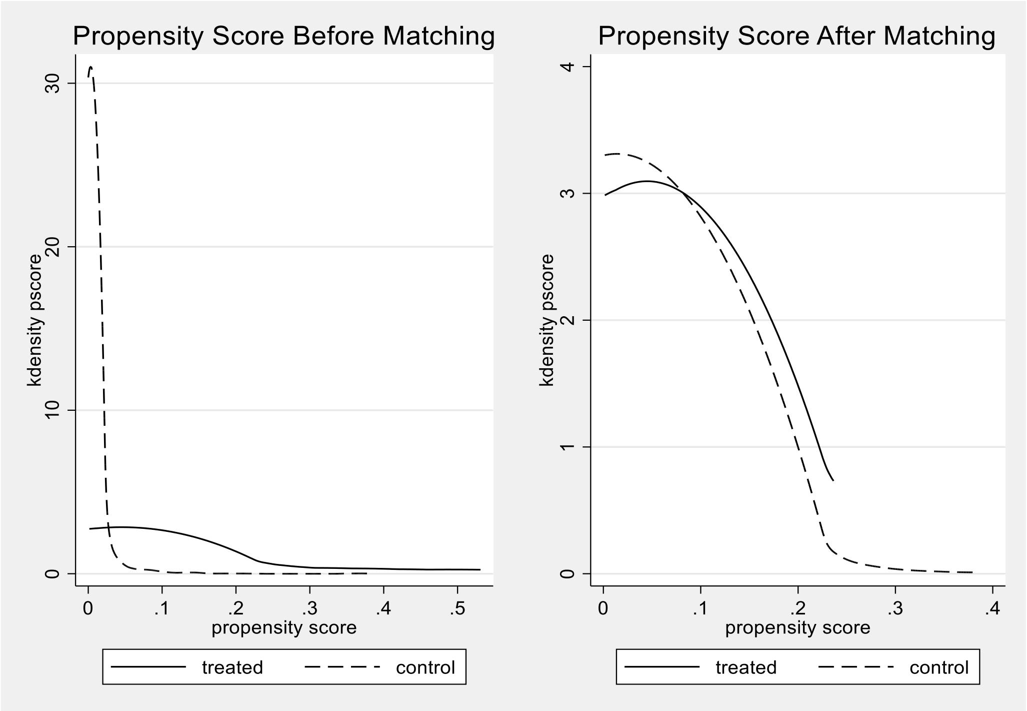

Supplementary Figure 2. Kernel density of treatment and control group

Notes: a. The left figure denotes the propensity score kernel density of treatment group and control group without matching. The right figure denotes the propensity score kernel density of treatment group and control group after matching. We used the same control variables as the baseline regression as matching covariates. b. Source data are provided in the Source Data file.

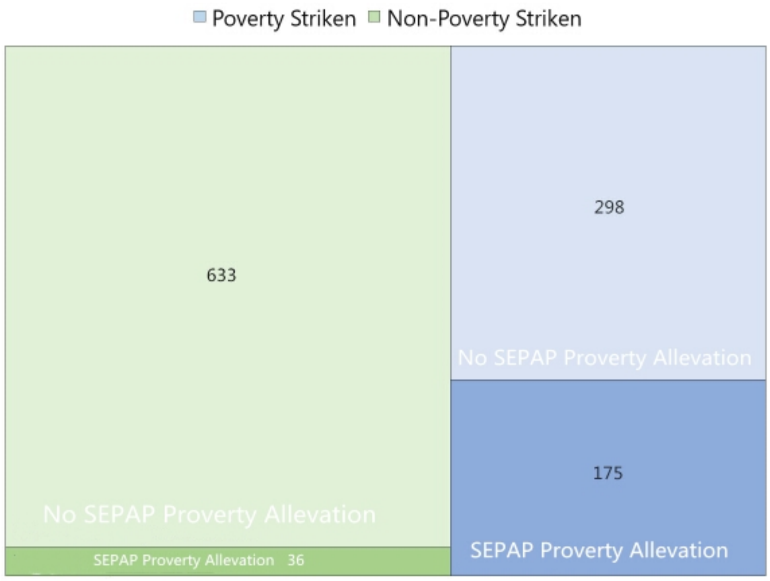

Supplementary Figure 3. Number of counties by type in our sample

Notes: a. The color depth indicates the different types of counties. 36 pilot counties for PV poverty alleviation are not state-level poverty-stricken. A total of 175 counties are not only pilot counties for PV poverty alleviation but are also state-level poverty-stricken counties, and 298 counties are only state-level poverty-stricken counties. 633 counties are not designated as state-level poverty-stricken counties and PV poverty alleviation pilot ones. b. Source data are provided in the Source Data file.

Supplementary Table 1. Partial list of factors affecting solar deployment or solar poverty alleviation

Authors	Year	Country or Region	Factors affecting solar deployment or solar poverty alleviation
Baurzhan and Jenkins	2016	Sub-Saharan African countries	Subsidies
Laufer and Schäfer	2011	Sri Lanka	Financing strategies
Obeng and Evers	2009	Ghana	Costs and market barriers, subsidies, stakeholders involvement, political and policy implications
Kamalapur et al.	2011	India	The quality of goods of SPV technology, cost, countrywide network, and restructured rural electric cooperatives, etc.
Yadav et al.	2019a, 2019b	India	Diversity in technology use, localised implementation and centralised planning and enforcement
Biswas et al.	2004	Bangladesh	Microcredit and subsidies
Adeoti et al.	2001	Nigeria	Fund research, development and demonstration; provide a subsidized price for more efficient light resources
Munro and Bartlett	2019	Uganda	Complex entanglements with different and changing energy technologies and institutional arrangements
Mandelli et al.	2016	Uganda	Load profiles
Rodríguez et al.	2018	Spain	Optimal PV potential strategies and thermal comfort strategies
Rosas-Flores et al.	2019	Mexico	Financial mechanisms; a new legal concept that supports mainly the development of financial instruments
Geall et al.	2018	China	Appropriate incentives for local officials and non-state actors
Li et al. , Xu et al., and Wu et al.	2019	China	Fund
Xue, Zhou and Liu	2017, 2018	China	Photovoltaic poverty alleviation industry
Li	2019	China	Subsidies, renewable energy quotas, electricity price and local consumption ratio, costs, energy storage and energy network technologies

Notes：Within the literature, Supplementary Table 1 summarizes the literature studying the factors affecting solar deployment or solar interventions for poverty alleviation.

Supplementary Table 2. Summary statistics

	Mean	S.D.	Q25	Median	Q75	N
County GDP Per Capita (ten thousand yuan)	2.849	1.852	1.654	2.355	3.410	4,566
County Savings Per Capita (ten thousand yuan)	1.895	0.960	1.260	1.699	2.278	4,566
Disposable Income for Rural Populations (ten thousand yuan)	0.914	0.298	0.686	0.878	1.122	3,783
SEPAP	0.007	0.081	0.000	0.000	0.000	4,568
Faction of Manufacturing Industries' GDP	0.442	0.147	0.348	0.448	0.540	4,562
Public Expenditure–to-Revenue Ratio	5.490	4.736	2.535	4.136	6.522	4,545
City-level Sun Hours (ten thousand hours/year)	0.183	0.055	0.142	0.181	0.220	4,356
Agriculture Land(million hectares)	0.147	0.324	0.008	0.031	0.129	4,440
Fraction of Middle School Students in Total Populations	0.044	0.012	0.036	0.043	0.051	4,564
Marketization Index	6.482	1.328	5.660	6.470	7.080	4,296
Provincial GDP per Capita (ten thousand yuan)	4.368	1.413	3.498	3.970	4.763	4,568

Supplementary Table 3. Correlation matrix

	DISINRURAL	SEPAP	SECONDGDPR	PUBEXINR	SUNHOUR	AGACRE	EDUCATION	MKTINDEX	GDPPROVINCE
DISINRURAL	1.00								
SEPAP	0.03	1.00							
SECONDGDPR	0.29***	-0.09***	1.00						
PUBEXINR	-0.44***	0.15***	-0.47***	1.00					
SUNHOUR	-0.18***	-0.00	0.02	0.06***	1.00				
AGACRE	0.19***	-0.02	0.03**	-0.12***	0.09***	1.00			
EDUCATION	-0.10***	0.07***	-0.03**	-0.04***	0.01	-0.04***	1.00		
MKTINDEX	0.58***	-0.04**	0.19***	-0.30***	-0.36***	0.24***	-0.23***	1.00	
GDPPROVINCE	0.48***	-0.06***	0.14***	-0.21***	0.07***	0.31***	-0.30***	0.72***	1.00

Notes: DISINRURAL represents rural disposable income. SEPAP represents whether or not a county was selected for the photovoltaic poverty alleviation policy in a specific year. SECONDGDPR depicts a proportion of the added value of the secondary industry to GDP. PUBEXINR shows the ratio of public expenditure to revenue. LNAGACRE examines the land used for facility agriculture. EDUCATION estimates the ratio of number of secondary school students to the total population. MKTINDEX represents marketization index. LNSUNHOUR indicates sunlight exposure time. LNGDPPROVINCE is used to investigate the per capita GDP of the province where the county is located.

Supplementary Table 4. Variance inflation factor test

Variable	VIF
SECONDGDPR	1.39
PUBEXINR	1.58
LN(AGACRE)	1.16
EDUCATION	1.35
MKTINDEX	2.88
LN(SUNHOUR)	1.42
LN(GDPPROVINCE)	2.78
SEPAP	1.01

Supplementary Table 5. Alternative definitions and regression specifications

	(1)	(2)	(3)
	Ln(DISINRURAL)	Ln(DISINRURAL)	Ln(DISINRURAL)
SEPAP (Poor and SEPAP Counties=1)	0.0261**		
	(2.57)		
SEPAP(All SEPAP Counties=1)		0.0272***	
		(2.82)	
SEPAP			0.0724***
			(9.27)
SECONDGDPR	0.0153	0.0100	0.0091
	(0.50)	(0.36)	(0.30)
PUBEXINR	-0.0037***	-0.0039***	-0.0037***
	(-3.39)	(-3.53)	(-3.53)
LN(AGACRE)	0.0004	0.0004	0.0006
	(0.20)	(0.25)	(0.35)
EDUCATION	0.1267	0.1326	0.1592
	(0.57)	(0.59)	(0.72)
MKTINDEX	0.0619***	0.0658***	0.0641***
	(6.94)	(7.34)	(7.12)
LN(SUNHOUR)	0.0576***	0.0633***	0.0659***
	(4.55)	(5.03)	(5.31)
LN(GDPPROVINCE)	-0.0096	-0.0245	-0.0461
	(-0.26)	(-0.68)	(-1.36)
County FE	Y	Y	Y
Year FE	Y	Y	Y
Region-Year FE	N	N	Y
Observations	3,144	3,203	3,203
Number of Counties	842	857	857
Adjusted R^2	0.06	0.06	0.06

Notes: The dependent variable is the natural logarithm of disposable income of rural people per capita. SEPAP represents whether or not a county was selected for the photovoltaic poverty alleviation policy in a specific year. SECONDGDPR depicts a proportion of the added value of the secondary industry to GDP. PUBEXINR shows the ratio of public expenditure to revenue. LN(AGACRE) examines the land used for facility agriculture facility agriculture land. EDUCATION estimates the ratio of number of secondary school students to the total population. MKTINDEX represents marketization index. LN(SUNHOUR) indicates sunlight exposure time. LN(GDPPROVINCE) is used to investigate the per capita GDP of the province where the county is located. ***, **, and * represent the significance levels of 1%, 5% and 10%, respectively. T-statistics are reported in parentheses.

Supplementary Table 6. Alternative definitions and regression specifications (excluding national poor counties)

	Exclude Counties that are both SEPAP and Poor	Exclude All Poor Counties
	(1)	(2)
	Ln(DISINRURAL)	Ln(DISINRURAL)
SEPAP	0.0829***	0.0874***
	(10.57)	(10.90)
SECONDGDPR	0.0139	-0.0417
	(0.42)	(-0.98)
PUBEXINR	-0.0030***	-0.0024**
	(-2.71)	(-2.13)
LN(AGACRE)	0.0001	0.0011
	(0.07)	(0.55)
EDUCATION	0.3256	0.7783***
	(1.35)	(2.76)
MKTINDEX	0.0468***	0.0425***
	(5.45)	(3.78)
LN(SUNHOUR)	0.0317**	0.0060
	(2.41)	(0.38)
LN(GDPPROVINCE)	0.0453	-0.0417
	(1.17)	(-1.19)
County FE	Y	Y
Year FE	Y	Y
Observations	2,587	1,798
Number of Counties	700	478
Adjusted R^2	0.04	0.04

Notes: The dependent variable is the natural logarithm of disposable income of rural people per capita. SEPAP represents whether or not a county was selected for the photovoltaic poverty alleviation policy in a specific year. SECONDGDPR depicts a proportion of the added value of the secondary industry to GDP. PUBEXINR shows the ratio of public expenditure to revenue. LN(AGACRE) examines the land used for facility agriculture land. EDUCATION estimates the ratio of number of secondary school students to the total population. MKTINDEX represents marketization index. LN(SUNHOUR) indicates sunlight exposure time. LN(GDPPROVINCE) is used to investigate the per capita GDP of the province where the county is located. ***, **, and * represent the significance levels of 1%, 5% and 10%, respectively. T-statistics are reported in parentheses.

Supplementary Table 7. The effect of PV policy on rural per capita disposable income (parallel trend test)

	(1)<br>Ln(DISINRURAL)	(2)<br>Ln(DISINRURAL)
SEPAP*(Year=-3)	0.0221	0.0282
	(1.20)	(1.52)
SEPAP*(Year=-2)	0.0372	0.0357
	(1.40)	(1.42)
SEPAP*(Year=-1)	-0.0136	-0.0125
	(-1.07)	(-1.05)
SEPAP*(Year=0)	0.0957***	0.0874***
	(3.86)	(3.66)
SEPAP*(Year=+1)	0.1951***	0.0960***
	(3.32)	(3.54)
SEPAP*(Year=+2)	0.2753***	0.2133***
	(8.87)	(7.05)
SEPAP*(Year=+3)	0.2850***	0.1972***
	(8.29)	(6.38)
SECONDGDPR		0.0115
		(0.39)
PUBEXINR		-0.0035***
		(-3.37)
LN(AGACRE)		0.0001
		(0.08)
EDUCATION		0.1737
		(0.79)
MKTINDEX		0.0608***
		(6.92)
LN(SUNHOUR)		0.0397***
		(3.13)
LN(GDPPROVINCE)		0.0170
		(0.45)
County FE	Y	Y
Year FE	Y	Y
Observations	3,756	3,203
Number of Counties	980	857
Adjusted R2	0.03	0.10

Notes: The dependent variable is the natural logarithm of disposable income of rural people per capita. SEPAP represents whether or not a county was selected for the photovoltaic poverty alleviation policy in a specific year. SECONDGDPR depicts a proportion of the added value of the secondary industry to GDP. PUBEXINR shows the ratio of public expenditure to revenue. LN(AGACRE) examines the land used for facility agriculture facility agriculture land. EDUCATION estimates the ratio of number of secondary school students to the total population. MKTINDEX represents marketization index. LN(SUNHOUR) indicates sunlight exposure time. LN(GDPPROVINCE) is used to investigate the per capita GDP of the province where the county is located. ***, **, and * represent the significance levels of 1%, 5% and 10%, respectively. T-statistics are reported in parentheses.

Supplementary Table 8. The effect of PV policy on rural per capita disposable income (sub-samples)

	Region		Economic Condition	
	(1)	(2)	(3)	(4)
	West	East	Poor	Rich
SEPAP	0.0585***	0.0716***	0.0475***	0.0013
	(6.39)	(4.82)	(4.38)	(0.59)
SECONDGDPR	0.0022	0.0260	0.0140	-0.0116
	(0.05)	(0.65)	(0.33)	(-0.27)
PUBEXINR	-0.0030**	0.0093***	-0.0032***	-0.0039*
	(-2.51)	(2.96)	(-2.88)	(-1.93)
LN(AGACRE)	0.0063	0.0020	0.0006	0.0018
	(1.29)	(0.57)	(0.28)	(0.68)
EDUCATION	0.4825	0.6853*	-0.2874	0.6711**
	(1.50)	(1.81)	(-0.94)	(2.01)
MKTINDEX	0.1390***	0.0495***	0.1059***	0.0420***
	(7.40)	(5.34)	(4.56)	(5.05)
LN(SUNHOUR)	0.2717***	0.0141	0.0985***	0.0270
	(6.84)	(0.52)	(5.72)	(1.35)
LN(GDPPROVINCE)	-0.2515***	-0.5355***	-0.0107	-0.1049**
	(-4.44)	(-4.20)	(-0.22)	(-2.05)
County FE	Y	Y	Y	Y
Year FE	Y	Y	Y	Y
Observations	1,106	902	1,437	1,683
Number of Counties	295	240	407	471
Adjusted R^2	0.18	0.15	0.11	0.03

Notes：The eastern region includes Liaoning, Beijing, Tianjin, Hebei, Shandong, Jiangsu, Shanghai, Zhejiang, Guangdong, Fujian and Hainan provinces; and the western region includes Xinjiang, Gansu, Qinghai, Inner Mongolia, Ningxia, Shaanxi, Sichuan, Chongqing, Guizhou, Yunnan and Guangxi provinces. The dependent variable is the natural logarithm of disposable income of rural people per capita. SEPAP represents whether or not a county was selected for the photovoltaic poverty alleviation policy in a specific year. SECONDGDPR depicts a proportion of the added value of the secondary industry to GDP. PUBEXINR shows the ratio of public expenditure to revenue. LN(AGACRE) examines the land used for facility agriculture facility agriculture land. EDUCATION estimates the ratio of number of secondary school students to the total population. MKTINDEX represents marketization index. LN(SUNHOUR) indicates sunlight exposure time. LN(GDPPROVINCE) is used to investigate the per capita GDP of the province where the county is located. ***, **, and * represent the significance levels of 1%, 5% and 10%, respectively. T-statistics are reported in parentheses.

Supplementary Table 9. The effect of PV policy on substitution dependent poverty variables

	(1)	(2)
	Ln(GDP Per Capita)	Ln(Saving Per Capita)
SEPAP	0.0271	-0.0878
	(1.48)	(-0.85)
SECONDGDPR	-0.0822	0.0765
	(-0.33)	(0.49)
PUBEXINR	-0.0104**	-0.0029
	(-2.34)	(-1.31)
LN(AGACRE)	0.0088**	-0.0033
	(2.23)	(-0.73)
EDUCATION	-0.0447	2.2705***
	(-0.07)	(3.25)
MKTINDEX	-0.0177*	0.0006
	(-1.76)	(0.05)
LN(SUNHOUR)	-0.0976**	-0.0142
	(-2.50)	(-0.43)
LN(GDPPROVINCE)	0.7741***	-0.0580
	(8.35)	(-0.71)
County FE	Y	Y
Year FE	Y	Y
Observations	3,896	3,896
Number of Counties	1,000	1,000
Adjusted R^2	0.07	0.01

Notes: The dependent variables are the natural logarithm of GDP per capita and saving per capita, respectively. SEPAP represents whether or not a county was selected for the photovoltaic poverty alleviation policy in a specific year. SECONDGDPR depicts a proportion of the added value of the secondary industry to GDP. PUBEXINR shows the ratio of public expenditure to revenue. LN(AGACRE) examines the land used for facility agriculture facility agriculture land. EDUCATION estimates the ratio of number of secondary school students to the total population. MKTINDEX represents marketization index. LN(SUNHOUR) indicates sunlight exposure time. LN(GDPPROVINCE) is used to investigate the per capita GDP of the province where the county is located. ***, **, and * represent the significance levels of 1%, 5% and 10%, respectively. T-statistics are reported in parentheses.

Supplementary Table 10. The effect of PV policy on raw rural per capita disposable income

	(1)	(2)
	DISINRURAL	DISINRURAL
SEPAP	0.0276*	0.0353***
	(1.95)	(2.66)
SECONDGDPR	0.0378	-0.0007
	(1.55)	(-0.03)
PUBEXINR	-0.0022***	-0.0031***
	(-2.78)	(-3.87)
LN(AGACRE)	-0.0014	0.0001
	(-0.81)	(0.07)
EDUCATION	0.7634***	0.6923***
	(3.83)	(3.47)
MKTINDEX		0.0418***
		(4.13)
LN(SUNHOUR)		-0.0089
		(-0.60)
LN(GDPPROVINCE)		0.1249***
		(3.71)
County FE	Y	Y
Year FE	Y	Y
Observations	3,598	3,203
Number of Counties	963	857
Adjusted R^2	0.01	0.05

Notes: The dependent variable is the disposable income of rural people per capita. SEPAP represents whether or not a county was selected for the photovoltaic poverty alleviation policy in a specific year. SECONDGDPR depicts a proportion of the added value of the secondary industry to GDP. PUBEXINR shows the ratio of public expenditure to revenue. LN(AGACRE) examines the land used for facility agriculture facility agriculture land. EDUCATION estimates the ratio of number of secondary school students to the total population. MKTINDEX represents marketization index. LN(SUNHOUR) indicates sunlight exposure time. LN(GDPPROVINCE) is used to investigate the per capita GDP of the province where the county is located. ***, **, and * represent the significance levels of 1%, 5% and 10%, respectively. T-statistics are reported in parentheses.

Supplementary Table 11. Endogenous treatment effect

	(1) Ln(DISINRURAL)	(2) Ln(DISINRURAL)
SEPAP	0.1471^{***}	0.1630^{***}
	(3.09)	(2.99)
SECONDGDPR	0.3331^{***}	0.3332^{***}
	(5.29)	(5.29)
PUBEXINR	-0.0167^{***}	-0.0167^{***}
	(-6.75)	(-6.75)
LNAGACRE	0.0302^{***}	0.0302^{***}
	(6.75)	(6.75)
EDUCATION	1.5658^{**}	1.5647^{**}
	(2.28)	(2.28)
MKTINDEX	0.0701^{***}	0.0701^{***}
	(8.77)	(8.77)
LNSUNHOUR	-0.0549^{*}	-0.0551^{*}
	(-1.89)	(-1.89)
LNGDPPROVINCE	0.2101^{***}	0.2106^{***}
	(5.87)	(5.89)
Equation for treatment effects		
SEPAP		
LNSUNHOUR	2.2861^{***}	1.2863^{***}
	(2.79)	(3.22)
LNGDPPROVINCE		-1.6510^{***}
		(-2.63)
County FE	Yes	Yes
Year FE	Yes	Yes
Observations	3,211	3,211
Number of Counties	865	865
Log Likelihood	-0.24	5.42

Notes: The dependent variable is the natural logarithm of disposable income of rural people per capita. SEPAP represents whether or not a county was selected for the photovoltaic poverty alleviation policy in a specific year. SECONDGDPR depicts a proportion of the added value of the secondary industry to GDP. PUBEXINR shows the ratio of public expenditure to revenue. LN(AGACRE) examines the land used for facility agriculture facility agriculture land. EDUCATION estimates the ratio of number of secondary school students to the total population. MKTINDEX represents marketization index. LN(SUNHOUR) indicates sunlight exposure time. LN(GDPPROVINCE) is used to investigate the per capita GDP of the province where the county is located. ***, **, and * represent the significance levels of 1%, 5% and 10%, respectively. T-statistics are reported in parentheses.

Supplementary Note 1. Robustness check- Subsample analysis

PV poverty-alleviation pilot counties are currently concentrated in the western, eastern, and central regions. The average county level GDP per capita, per capita savings deposit-balance, per capita rural disposable income, and other important indicators reflecting the income level of residents (or farmers) in China's three major regions descend from east to west. Thus, the economic development of different regions has a ladder distribution. Poverty alleviation funds distribution discipline may be greater in areas with high income where the system is established and the marketization level is high. Moreover, high-income regions are also characterized by effective data/information sharing and cross-sectoral connectivity that extends to farmers and the agricultural sector. However, precisely because of the high levels of regional economic development, central and local governments may be inclined to support low-income counties with funds and technical support, thereby reducing the poverty alleviation efforts of high-income counties.

In addition to difference in regional economic development, natural features impact the mode of PV poverty alleviation in different parts of China. Eastern parts of China are suitable for small-scale distributed PV power stations and rapid construction on small parcels of land. This mode has comparatively low economies of scale. The less developed western part of China, with its desert, barren mountains, and open spaces is conducive to centralized large-scale grid-connected PV installations. This mode requires large-scale investment, long construction periods, and large tracts of land. It thus enjoys comparative economies of scale, which may have further poverty alleviating effects. The regional impact of targeted PV poverty alleviation policies is therefore complex. The following hypotheses are proposed after the synthesis of this study:

H4: The effect of PV poverty-alleviation policy is higher in areas with higher per capita income levels.

H5: The effect of PV poverty-alleviation policies is greater in the eastern parts of China.

We analyze the poverty alleviation effects of PV poverty alleviation policies by subsamples according to regional and economic conditions, in which the regional grouping focuses on comparing the east and the west, and the economic conditions refer to the county GDP per capita. The results in supplementary table 8 show that the coefficient of PV poverty alleviation policy on rural per capita disposable income in the eastern and western regions is positive and statistically significant. The coefficient of PV poverty alleviation policy variables in the east is 0.0716, which is slightly higher than 0.0585 in the western region. According to the per capita GDP value, our sample is divided into two categories: relatively rich areas and poverty-stricken areas. Empirical results reveal that the PV poverty alleviation policy has a greater effect on the poor regions, and the effect amounts to 4.86%. In rich counties, poverty alleviation has no significant results with a coefficient of 0.0013 and fails to pass the significance test. Combining the classifications, relatively poor places are more likely to alleviate poverty from the PV project policy. The reason why the PV policy in the eastern region has a significant effect on poverty alleviation may be that some eastern counties have lower county-level per capita GDP than those in the western region, although the overall economic level of the eastern region is relatively rich. In addition, the complete establishment of the local system and the rational use of poverty alleviation resources in eastern regions also make the PV poverty alleviation policy effective.

Supplementary Note 2. Robustness check- Measures of dependent variables

The model is re-estimated by re-measuring the dependent variables using local per capita GDP and resident per capita savings deposit balance. However, the impact of PV policy on these two variables was not significant (see supplementary table 9). The possible reason is that the purpose of PV poverty

alleviation is to alleviate the poverty of farmers. However, the above two variables reflect the per capita income of the entire county, including urban residents. Therefore the per capita disposable income in rural areas is significantly affected by PV policies but not by these two alternative measures. This result reflects that the poverty reduction effect is due to PV intervention rather than other reasons, as other policy interventions may have a significant impact on the two alternative dependent variables. We also use the absolute value measure of rural per capita disposable income to re-test. Supplementary table 10 provides the robust results: the implementation of PV poverty alleviation policy can increase rural per capita disposable income by 353 yuan/year.

Supplementary Note 3. Robustness check- Endogenous treatment effect

We estimate a linear regression with endogenous treatment effects. The natural logarithm of sunlight hours and per capita GDP of the province where the county is located. These two variables are highly correlated with the selection of the SEPAP in China. Supplementary table 11 presents the relevant results.

In Column (1), the estimated coefficient of SEPAP amounts to 0.1471, which is significant at the 1% level. The economic magnitude of the treatment effect is twice of that in the baseline results without accounting for the endogenous treatment effects, indicating potential underestimate of the effect of SEPAP on rural household income in the baseline model. The result of treatment effects equation shows that sunlight hours are significantly positively correlated with the likelihood of being selected into the SEPAP. Column (2) adds natural logarithm of provincial per capita GDP into the equation for treatment effects, and the coefficient of SEPAP increases to 0.1630, which is significant at the 1% level. The result of treatment effects equation shows that provincial per capita GDP is negatively correlated with the likelihood of being selected into the SEPAP. The result is also consistent with our conjecture since the SEPAP is partially determined by the local economic conditions.
